# Age at recognition and age at presentation for surgery for congenital and developmental cataract in Kazakhstan

**DOI:** 10.1080/07853890.2022.2091156

**Published:** 2022-07-14

**Authors:** Aliya Kabylbekova, Serik Meirmanov, Altyn Aringazina, Lukpan Orazbekov, Ardak Auyezova

**Affiliations:** a Department of Population Health and Social Science, Kazakhstan's Medical University “KSPH”, Almaty, Kazakhstan; b College of Asia Pacific Studies, Ritsumeikan Asia Pacific University, Beppu City, Japan; c Caspian International School of Medicine, Caspian University, Almaty, Kazakhstan; d Department of Pediatric Ophthalmology, Kazakh Eye Research Institute, Almaty, Kazakhstan

**Keywords:** Childhood cataract, children, cataract surgery, delay, Kazakhstan

## Abstract

**Purpose:**

To investigate the age at recognition and presentation for surgery for congenital and developmental cataract at Kazakh Eye Research Institute in Kazakhstan.

**Methods:**

A retrospective review of children aged 0–18 years, who presented with congenital and developmental cataract between January 1, 2010 and December 31, 2020. All medical records were reviewed. Gender, age at recognition, age at surgery, laterality, residential location (rural/urban) were recorded.

**Results:**

The study population included 897 patients of children presented with congenital and developmental cataract over a 10-year study period, 58% of them were boys and 44.6% were from rural areas. Cataract was bilateral in 621 (69.2%) and unilateral in 276 (30.8%) of patients. Median age at recognition for patients with congenital/developmental cataract was 12 months. Median age at surgery for congenital/developmental cataract was 51 months. Only 14.7% of children underwent surgery within first year of life. The urban citizens underwent surgery earlier than patients from rural areas. The median delay in presentation for surgery was 15 months.

**Conclusion:**

The average age at cataract surgery in the population of Kazakhstan is much older than in developed countries. It is essential to study barriers that associated with delayed presentation to build strategies to overcome them.

**Key messages**
- It is known that cataract surgery in children early in life provides favourable visual outcome.
- Children with congenital and developmental cataract in Kazakhstan experience delay in surgical treatment.
- Children from rural areas undergo cataract surgery later than urban citizens.

## Introduction

Cataract is a leading cause of avoidable childhood blindness worldwide [1]. The global prevalence of childhood cataract is estimated to be from 0.32 to 22.9 per 10,000 children, while prevalence of congenital cataract ranges from 0.63 to 9.74 per 10,000 children [2]. Epidemiologic data is mostly focussed on congenital cataracts [3].

In many countries, use the clinical classification of cataract in children according to the age of onset [4,5]. Paediatric cataract is called congenital, if presents during first year of life, and developmental, if occurs later in early childhood [4,6–8].

Lens opacity in infancy and early childhood causes deprivation amplyopia, which disrupts the child’s educational and psychosocial development [5]. It was established that unilateral visual deprivation inhibits developing visual system more severely than bilateral deprivation due to the competition of affected eye with an unaffected one [9].

The main treatment option of childhood cataracts is surgical operation. Age at surgery is the key modifiable factor in determining visual outcome: the later the surgery is performed, the worse is the visual outcome [10,11]. It has been shown in IoLunder2 prospective cohort study that children with bilateral cataract, operated in the third month of life, showed a 4 logMAR line worse vision, than children, who underwent surgery in the first month of life [11]. Previous studies reported that unilateral congenital cataract surgery is most effective during first 6 to 8 weeks after birth, [9] and within the first 14 weeks, for bilateral cataract [12]. Some morphologic types of developmental cataracts also require timely intervention due to the rapid progression [13].

Early detection of lens opacity makes possible to perform early surgical intervention [14]. In developed countries, the presence of red reflex screening programmes of newborns and infants allows providing early identification and therefore timely surgery [15]. In Sweden, eye screening provides detection up to 64% of congenital cataract cases in maternity ward [15]. Seventy-nine percent of Swedish children with congenital cataract were referred to ophthalmologist within 42 days by maternity wards or well baby clinic and 89% of children within 100 days [14]. However, it has been reported that in United Kingdom only 47% of children with congenital cataract were detected through screening by 3 months of age [16]. In developing countries with no routine neonatal eye screening cataract is detected much later [5]. In India, 12.4% of children with childhood cataract were recognised within 28 days of life [5]. In China, 51% of children were diagnosed to have cataract at the age older than 3 years of life [7].

Previous studies from developing countries reported delay in presentation for surgery of children with congenital and developmental cataract [5–7]. There are different barriers, including gender inequality, low paediatric cataract awareness, availability of health care services and others, that result in surgical delay [17,18].

The objective of this study was to determine the age at recognition and at presentation for cataract surgery, delay in presentation for surgery, as well as epidemiological characteristics of congenital and developmental cataracts seen at the Kazakh Eye Research Institute over the past 10 years.

## Methods

This study was approved by the Institutional Review Board of the Kazakhstan’s Medical University “KSPH”, Almaty, Kazakhstan (number IRB-141 from May 31, 2021, outgoing number 04-09-237 from May 31, 2021) and conformed to the guidelines of the Declaration of Helsinki. We retrospectively reviewed medical records of all patients with congenital and developmental cataract aged 0 to 18 years who underwent primary cataract surgery at Kazakh Eye Research Institute in Almaty, Kazakhstan from January 1, 2010 to December 31, 2020. Kazakh Eye Research Institute is the only tertiary eye care centre providing surgery for children with congenital and developmental cataract in Kazakhstan. Cases of congenital and developmental cataract (code Q 12.0 in International Classification of Diseases 10) were identified using the database of the Statistics Department of the Kazakh Eye Research Institute. We excluded cases with missing or incomplete data, as well as cataracts with traumatic, acquired systemic (e.g. diabetes), or acquired ocular aetiology (e.g. uveitis). Data on remaining 897 patients were used for the subsequent analyses.

### Case definitions

In clinical practice in Kazakhstan, cataracts in children are classified as congenital (code Q 12.0 in International Classification of Diseases 10), with the exception of secondary forms of the lens opacity due to trauma, acquired systemic or ocular pathology. Due to the fact that it is challenging to distinguish retrospectively between congenital and developmental cataracts, both of these clinical categories were combined in the present study and presented as congenital/developmental cataract.

### Age at recognition and at surgery

Age at which the eye problem was recognised by caregivers was defined as age at recognition. Age at which the child underwent cataract surgery was defined as age at surgery. In cases of bilateral cataract the age at first eye’s surgery was used for analysis.

### Delay of presentation

Delay in presentation for surgery was defined as the median number of months between recognition and presentation for cataract surgery.

### Collection of data

All the eligible medical charts were carefully reviewed. We recorded the following data from the medical histories of these patients: gender, age at recognition, age at surgery, residential location (rural/urban), laterality.

### Statistical analysis

The data were analysed using StatTech v. 2.4.3 (Developer - StatTech LLC, Russia). Quantitative variables were assessed for normality using the Kolmogorov–Smirnov test. Quantitative variables following non-normal distribution were described using median (Me) and interquartile range (IQR). Categorical data were described with absolute and relative frequencies. Mann–Whitney U-test was used to compare two groups on a quantitative variable whose distribution differed from the normal distribution. Z test of proportion was used to measure any statistical difference between two populations on a single categorical characteristic (gender). A *p* value less than 0.05 was considered statistically significant.

## Results

A total of 897 patients with congenital and developmental cataract were identified during the study period. Of the 897 patients, 520 (58.0%) were boys and 400 (44.6%) were from rural areas. The proportion of boys and girls undergoing cataract surgery was approximately equal (Z-value −0.0062, *p* = 0.992). Bilateral cataract was present in 621 (69.2%) children, and unilateral cataract was present in 276 (30.8%) children. According to the study definition, the demographic data of patients by laterality are shown in Table 1.

**Table 1.** Demographic data by laterality.

Category	**Bilateral cataract** ***n* (%)**	**Unilateral cataract** ***n* (%)**	Total
Total patients	**621 (69.2)**	**276 (30.8)**	**897 (100)**
Gender			
Boys	**377 (60.7)**	**143 (51.8)**	**520 (58.0)**
Girls	**244 (39.3)**	**133 (48.2)**	**377 (42.0)**
Residental location			
Rural	**284 (45.7)**	**116 (42.0)**	**400 (44.6)**
Urban	**337 (54.3)**	**160 (58.0)**	**497 (55.4)**

The median age at recognition of congenital/developmental cataract was 12 months (IQR = 46 months), while the median age at surgery was 51 months (IQR = 70 months). Only 132 (14.7%) of children underwent cataract surgery within first year of life. The median delay in presentation for congenital/developmental cataract surgery was 15 months (IQR = 35 months) with 538 (60%) children having ≥ 12 months. The comparative data between unilateral and bilateral cases on the age at recognition, at surgery and the duration of delay is shown in Table 2.

**Table 2.** Age at recognition, age at surgery and delay interval before presentation for surgery of patients **with bilateral and unilateral cataract (months)**.

Category	Bilateral cataract	Unilateral cataract	*P* value
Median age at recognition	12.0 (IQR 46.00)	12.0 (IQR 48.0)	.067
Median age at surgery	52.0 (IQR 68.00)	48.0 (IQR 80.25)	.718
Median delay interval	16.0 (IQR 36.00)	13.0 (IQR 32.00)	.077

**Figure 1.**
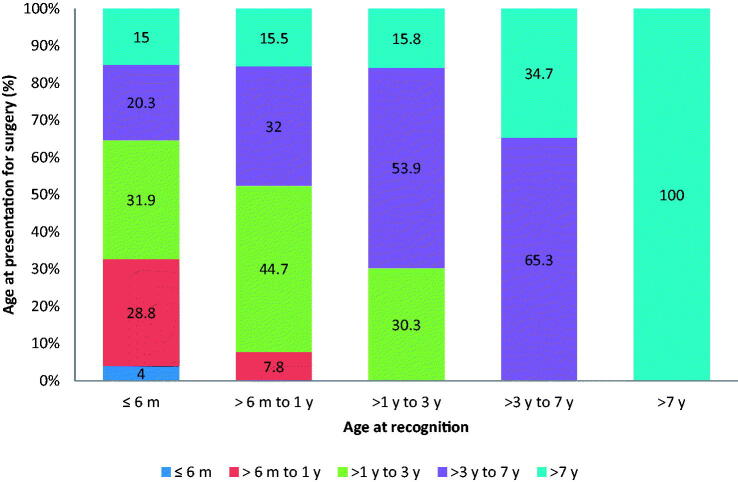
Comparison of age at recognition and the age at presentation for surgery for childhood cataract (Proportion of children, m: month; y: years).

In 379 (42.3%) of the children with congenital/developmental cataract, the condition was recognised within first 6 months of life. However, only 124 (32.8%) of these 379 children underwent surgery within first year from birth, 121 (31.9%) between 1 and 3 years, 77 (20.3%) between 3 and 7 years and 57 (15%) after 7 years of age (Figure 1).

We evaluated whether the gender had influenced the age at recognition and age at presentation for cataract surgery (Table 3). The median age at which congenital/developmental cataract was recognised was similar for boys and girls (*p* = .554) and both males and females were operated at a similar age (*p* = .402).

**Table 3.** Effect of gender on the age at recognition and age at surgery.

Category	Boys Median age (IQR)	Girls Median age (IQR)	*p* Value
Age at recognition (months)	12.0 (IQR 45.00)	12.0 (IQR 48.00)	.554
Age at surgery (months)	49.0 (IQR 65.50)	53.0 (IQR 77.00)	.402

Furthermore, we investigated the effect of residential location on the age at recognition and at presentation for surgery (Table 4). Patients from rural areas underwent surgery for congenital/developmental cataract at a much older age than urban citizens (*p* < .001), despite the similar age at recognition of congenital and developmental cataract (*p* = .235).

**Table 4.** Effect of residential location on age at recognition and age at surgery.

Category	Rural Median age (IQR)	Urban Median age (IQR)	*p* Value
Age at recognition (months)	12.0 (IQR 48.00)	12.0 (IQR 42.00)	.235
Age at surgery (months)	58.0 (IQR 76.25)	45.0 (IQR 63.00)	<.001

**Figure 2.**
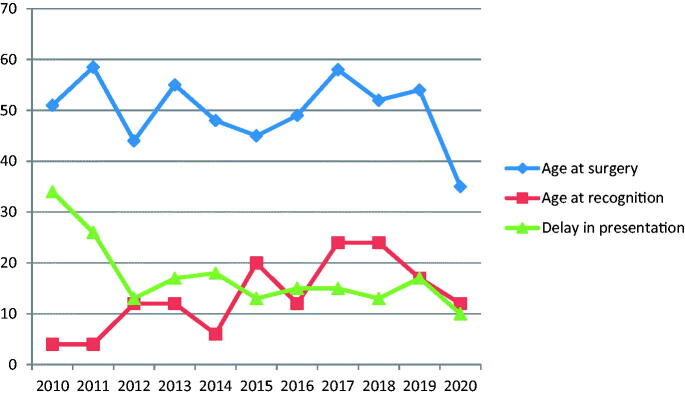
Dynamics of ages at recognition of cataract and at surgery, and duration of delay in presentation for surgery in congenital/developmental cataract cases (median number of months).

There were declining trends in the age at surgery and delay interval before presentation for surgery in patients with congenital/developmental cataracts from 2010 to 2020, but not in the age at recognition (Figure 2).

## Discussion

The median age at surgery for congenital/developmental cataract was 51 months (about 4 years). The proportion of children undergoing surgery within 6 months from birth was lower (4%) in the current study than has been reported in China (9.7%) [7] and in India (16%) [5]. Zhu *et al.* [19] reported that the average age of children undergoing congenital/developmental cataract surgery in East China is about four and a half years, which is similar to our findings. These findings indicate that there are barriers to early surgery for congenital and developmental cataract in Kazakhstan.

Delay in presentation for surgery is common for developing countries [5–7]. The median delay in presentation for surgery in children with congenital/developmental cataract in our study was significantly longer (15 months) than delay in presentation for cataract surgery from developed country with maternity-ward eye screening protocol [15]. More than a half of children presented for surgery a year and later, after parents had noticed a cataract. This finding is comparable to recently reported finding from Tanzania where 52.5% of children underwent surgery with a lag time of ≥ 12 months [20]. Even though the age at surgery and delay for presentation for surgery is decreasing over time in our population, there is still need for implementation of health care strategies to ensure the timely surgical intervention to prevent the development of childhood blindness due to preventable reason.

We found that patients from rural areas present for surgery at much older age than urban citizens. It has been shown previously that barriers for cataract surgery exist at the health service, community and family level [17,21]. Possible reason for the delayed presentation for surgery of cataract patients from rural areas at the health service level may be the fact, that according to the reports of Bureau of National Statistics of the Republic of Kazakhstan, there are few paediatric ophthalmologists in our country and they work mostly in urban centres [22]. So, patients from remote areas probably face poor access to quality health care, which result in significant delay in presentation for surgery. Other possible explanation for urban–rural differences in timing of cataract surgery at community and family level may be due to the cultural and social determinants.

In the majority of cases (69.7%), children presented for congenital/developmental cataract surgery with bilateral cataract which was similar to 65.8% reported by Nagamoto *et al.* in Japan [23] and 71% reported by Fakhoury *et al.* in France [24]. However, it has been shown that globally the prevalence of bilateral and unilateral childhood cataract is found to be similar [2]. This would suggest that high proportion of unilateral cataract cases in our environment do not undergo surgical intervention probably because it affects overall development of the children much less than in bilateral cases.

It is well established that lens opacity in children should be removed surgically early on to prevent the development of irreversible deprivation amblyopia, especially in unilateral cases due to the competition between affected and unaffected eyes [9]. In our study, we found that children presented for cataract surgery at the similar age in unilateral and bilateral cases. The average delay in presentation for surgery was also comparable. We suggest that parents of children affected unilaterally may not seek appropriate eye care sooner because the child’s general development is overall normal.

Sheeladevi *et al.* reported that there is no gender difference in the prevalence of childhood cataract worldwide [2]. In our study, the proportion of boys and girls undergoing cataract surgery did not differ statistically, which was similar to the gender distribution in France (47 vs. 53%), [24] and in Sweden and Denmark (51.7% boys, 48.3% girls) [25].

It has been reported that there are gender inequalities in surgery for childhood cataract in many countries [26]. Possible reasons for it include cultural, economic and social context [26]. We found that girls present for congenital/developmental cataract surgery at older average age than boys although the difference is not statistically significant. This finding may suggest that the parents of girls with eye concerns may not seek timely quality surgical care and it is necessary to find out the hidden reasons for this in our population.

The main limitation of our study includes retrospective nature of the study. The categorisation of cataract in early childhood is challenging. The most common definition of congenital and developmental cataract is based on the age of onset: cataract is classified as congenital if present within 1 year of life or developmental, if present after infancy and is not due to trauma [4,6,7]. Due to the retrospective character of our study and the lack of a national red reflex screening program of newborns and infants, allowing determining the age, at which cataract is present, it was challenging for us to classify cases in our study and we decided to report findings in combined manner as congenital/developmental cataract.

Another limitation is the fact that we were unable to obtain information about socio demographic factors, such as age, educational status and occupation of parents, number of siblings, that were found to affect the age at presentation for surgery in previous studies [5,6,27].

In conclusion, this is the first report on the age at recognition and presentation for surgery of the children with congenital and developmental cataract over 10-year study period from Kazakhstan. The average age of the children with congenital and developmental cataract at surgery is much older than in developed countries. Future efforts should be taken to study the reasons of delayed presentation for surgery in the population of Kazakhstan to develop strategies to overcome these barriers. It is essential to establish national red reflex screening of newborns and infants for timely detection of sight-threatening ocular abnormalities like congenital cataract.

## Data Availability

The data that support the findings of this study are available on request from the corresponding author, [AK]. The data are not publicly available due to [restrictions e.g. their containing information that could compromise the privacy of research participants].
